# Report of two severe cases of Japanese encephalitis virus in travelers returning to France, 2023-2024: clinical, diagnostic, and public health implications

**DOI:** 10.1128/asmcr.00054-24

**Published:** 2025-01-07

**Authors:** Nazli Ayhan, Laura Pezzi, Hermann Do Rego, Michael Thy, Sandra Devatine, Giovanna Melica, Matthieu Gautier, Raphaelle Klitting, Ségolène Brichler, Guillaume André Durand, Xavier de Lamballerie, Gilda Grard

**Affiliations:** 1 National Reference Center for Arboviruses, Inserm-IRBA, Marseille, France; 2 Unité des Virus Émergents (UVE: Aix-Marseille Univ, Università di Corsica, IRD 190, Inserm 1207, IRBA) 128791 , Marseille, France; 3 Medical and Infectious Diseases ICU, Bichat Claude Bernard University Hospital, Université Paris Cité, AP-HP 60152 , Paris, France; 4 Service de Maladies Infectieuses et tropicales, CHU Henri Mondor, Assistance Publique–Hôpitaux de Paris, Créteil, France; 5 Inserm U955 Team 18, Institut Mondor de Recherche Biomédicale (IMRB), Créteil, France; 6 Infectious Diseases and Clinical Immunology Department, Henri Mondor Hospital 55471 , Créteil, France; 7 Service de Microbiologie Clinique, CHU Avicenne, Assistance Publique–Hôpitaux de Paris, Bobigny, France; Vanderbilt University Medical Center, Nashville, Tennessee, USA

**Keywords:** Japanese encephalitis virus, viral encephalitis, JEV, case report, neurotropic virus

## Abstract

**Background:**

Japanese encephalitis virus (JEV) is a mosquito-borne pathogen responsible for Japanese encephalitis (JE) clinical cases in Asia and the Western Pacific. Non-JE-vaccinated patients can develop potentially life-threatening neurologic forms.

**Case Summary:**

Here, we present two severe, non-fatal JEV cases in returning travelers from Cambodia (Case 1) and Cambodia and Vietnam (Case 2), imported to France in 2023–2024. Neither patient was vaccinated, and both presented neurologic symptoms requiring hospitalization. Cases were confirmed by RT-qPCR, IgM, and IgG rise and seroneutralization. Interestingly, this is the second report to describe the detection of the JEV genome in urine by RT-qPCR.

**Conclusion:**

This study highlights the critical importance of JE vaccination and the implementation of other personal protective measures to avoid mosquito bites when traveling to a JEV-endemic region.

## INTRODUCTION

Japanese encephalitis virus (JEV) is a mosquito-borne flavivirus that causes an estimated 50–100,000 clinical cases of Japanese encephalitis (JE) every year, including severe presentations with neurological complications and associated mortality (1–4). JE primarily affects children and young adults in endemic areas and is one of the most important causes of viral encephalitis worldwide (5, 6). While there is no effective treatment for JE, several licensed vaccines are available to prevent the disease (7).

JEV is transmitted between birds (the primary reservoir) and pigs (the major amplifying host) by *Culex* species mosquitoes; humans and horses are considered incidental, dead-end hosts (8–10). Historically present in Asia and the Western Pacific, JEV has recently caused an outbreak in mainland Australia (11).

Diagnosis of JE is challenging and often relies on serological assays, either the detection of anti-JEV immunoglobulin (Ig)M in cerebrospinal fluid (CSF) or serum samples, using enzyme-linked immunosorbent assay (ELISA) or seroneutralization assays (serology gold standard [WHO]). The low viral loads, short viremic window, and delay in the appearance of the neurological manifestations make JEV infection difficult to detect via molecular biology methods, with the detection of viral RNA in 0%–25% of clinical cases (12). Serum or plasma are the preferred samples for JEV molecular diagnosis, together with CSF in patients with JEV infections involving the central nervous system (13). Rarely, JEV has been detected by PCR in throat swabs, saliva, urine, and whole blood collected from patients in the early phase of the disease (14–16).

Here, we report two severe, non-fatal JEV cases in returning travelers from Southeast Asia.

## CASE PRESENTATION

### Case 1

A 29-year-old woman with no pre-existing medical conditions traveled to Cambodia from 10 December to 24 December 2023, primarily in Siem Reap (central Cambodia) and Koh Rong, an island in western Cambodia. She had not received the JE vaccination and reported no specific exposures apart from frequent mosquito bites during her stay. The day after returning to France, she began to experience symptoms, including headache on 25 December, fever on 26 December, and confusion on 27 December, as reported by her relatives. On 28 December, the patient was admitted to the emergency department of Saint-Joseph Hospital in Paris, France. On admission, the patient exhibited fever, headache, and neurological symptoms, including confusion, obtundation, grasping reflex, oculomotor abnormalities, and neck stiffness.

The blood test indicated the presence of an inflammatory syndrome, as evidenced by elevated levels of C-reactive protein (CRP) of 107 mg/L (normal range: <3 mg/L) and an increased white blood cell count (leukocytosis) of 13,000 × 10^6^/L (normal count: 4,000-11,000 × 10^6^/L). The lumbar puncture performed at admission revealed a CSF leukocyte count of 2,200/mm³ (normal count: ≤5/mm^3^), predominantly (82%) lymphocytes, along with a CSF erythrocyte count of 800/mm³ (normal count: 0), and a CSF protein concentration of 1.18 g/dL (normal range: 0.18–0.58 g/L), with no evidence of a raised CSF:serum glucose ratio on day 4 post-symptom onset.

The CSF Gram stain and PCR BIOFIRE FILMARRAY Meningitis/Encephalitis Panel (including herpes simplex virus and *Listeria monocytogenes*) tests were negative, as were the subsequent bacteriological culture results.

Cerebral computed tomography (CT) demonstrated no abnormalities. Cerebral magnetic resonance imaging (MRI) revealed FLAIR hypersignal in the left hippocampus, paramedian thalamus, and tegmentum (see Fig. S1 at https://github.com/LauraPezzi/JEV-imported-in-France-2024). The electroencephalogram (EEG) revealed a pathological trace of non-reactive encephalopathy, which did not indicate the presence of epilepsy.

**Fig 1:**
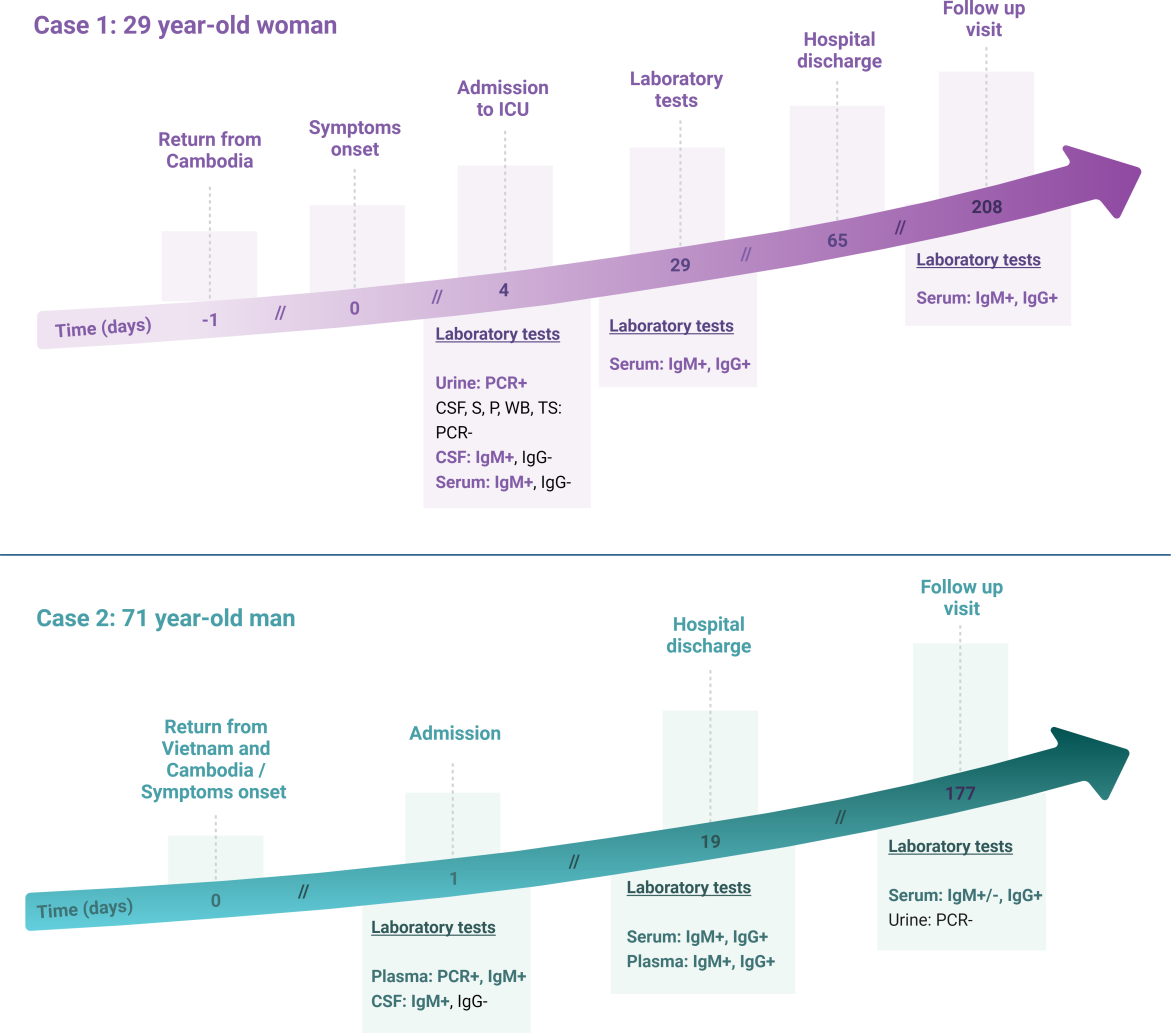
Timeline of JEV laboratory tests and clinical features of Cases 1 and 2. S, serum; P, plasma; WB, whole blood; TS, throat swab; and ICU, intensive care unit. Figure created with BioRender.com.

The patient was treated empirically with ceftriaxone (2 g × 2/day), amoxicillin (20 g/day), and acyclovir (700 mg × 3/day) intravenously and transferred to the neurology department. Ceftriaxone and amoxicillin were stopped after 24 hours due to the lack of bacteriological documentation.

On 29 December, the patient’s condition declined, resulting in a coma. She was transferred to the medical intensive care unit (ICU) at Bichat-Claude Bernard Hospital in Paris, intubated, and ventilated. The second lumbar puncture yielded the following results: 221 leukocytes/mm³ (including 81% lymphocytes), 33 erythrocytes/mm³, and 1.09 g of protein with no evidence of a raised CSF:serum glucose ratio. Gram stain and bacteriological culture were again negative, and because of a negative PCR FILMARRAY, acyclovir was stopped at 48 hours. The patient exhibited severe bradycardia, which was considered likely attributable to dysautonomia resulting from thalamic damage. This resulted in the necessity for the provisional placement of a transvenous pacemaker for a period of 4 days.

On 29 December (day 4 of symptoms), a number of samples were collected, including CSF, plasma, serum, whole blood, throat swab, and urine. JEV viral RNA was identified in urine by RT-qPCR assay using an in-house assay (12) on a Panther Fusion (HOLOGIC) (see the supplemental material at https://github.com/LauraPezzi/JEV-imported-in-France-2024), whereas all the other samples tested negative. The EUROIMMUN anti-JEV IgM and IgG ELISA kits were used to test serum and CSF samples; specific anti-JEV IgMs were detected in both, while the anti-JEV IgG test was negative. IgM testing against dengue (DENV) and West Nile virus (WNV) was found weakly positive in serum by ELISA EUROIMMUN anti-DENV and anti-WNV IgM kits; this was considered a cross-reactivity since (i) DENV and WNV ratios were five times lower than JEV ratio, (ii) IgG testing was negative for both viruses, and (iii) CSF sample was positive only for anti-JEV IgM (see Table S1 at https://github.com/LauraPezzi/JEV-imported-in-France-2024). Serum was also negative for IgM anti-chikungunya (CHIKV) and Zika virus (ZIKV) (ELISA EUROIMMUN kits), as well as by RT-qPCR for CHIKV, DENV, ZIKV, and WNV (in-house assays on Panther Fusion, HOLOGIC).

Seroconversion for anti-JEV IgG was observed in a convalescent serum sample collected 29 days after the onset of symptoms, as confirmed by a seroneutralization test. Laboratory methods and findings are presented in the supplemental material.

The patient’s condition improved, and she was discharged from the intensive care unit, transferred to the neurology unit, and subsequently to the rehabilitation unit. She returned to her residence on 1 March, 2 months after admission.

With the exception of attention deficits and pronounced asthenia, no sequelae have been observed. Tests on samples collected during a follow-up visit almost 7 months after symptom onset showed the persistence of anti-JEV IgM and IgG in serum (Fig. 1).

### Case 2

A 71-year-old man traveled to Vietnam and Cambodia from 9 January to 26 January 2024, and he indicated having been rarely bitten by mosquitoes during his stay. He went to the Mekong Delta and Hoi An in Vietnam; in Cambodia, he visited Siem Reap, Koh Rong, and the capital Phnom Penh. The patient was not vaccinated against JE and had no remarkable medical history. He traveled with his wife who experienced no symptoms.

Symptoms started just before traveling back to France on 26 January with fever and headache, and during the flight, aphasia appeared. He went directly to Mondor Hospital, Créteil, France, after landing. At admission, on 27 January (day 1 of symptoms), he had fever (38.6°C), headache, and central neurological involvement with aphasia and homonymous lateral hemianopia leading to transfer to ICU on 28 January.

The blood test indicated no inflammatory syndrome, normal white blood cell counts, and CRP at 5 mg/L (<3 mg/L: normal reference range). The initial lumbar puncture revealed a leukocyte count of 1,000/mm³ (normal CSF leukocyte counts are ≤5/mm^3^), predominantly neutrophils (63%) and lymphocytes (37%), and a protein concentration of 0.89 g/dL (0.18–0.58 g/L is the reference range).

As for Case 1, the Gram stain and PCR BIOFIRE FILMARRAY Meningitis/Encephalitis Panel provided negative results, as well as the bacteriological culture results. Cerebral CT and MRI revealed no anomalies. EEG showed severe slowing and poorly organized tracing without epileptic activity.

Antimicrobial treatment was introduced with cefotaxime 18 g/day, amoxicillin 24 g/day, and acyclovir 2.5 g/day on 29 January. Treatments were stopped when the CSF culture returned negatives. Neurological amelioration appeared on 31 January, and the patient was discharged from ICU on 3 February (Fig. 1).

JEV RNA was detected in a plasma sample collected at admission using the aforementioned automated RT-qPCR assay; plasma and CSF drawn on the same day were positive for anti-JEV IgM (EUROIMMUN ELISA kit) (see Table S1 at https://github.com/LauraPezzi/JEV-imported-in-France-2024). As for Case 1, a weakly positive result for IgM DENV and WNV was observed; again, it was considered a non-specific serological reaction due to cross-reactivity between flaviviruses. The same previously explained procedure was taken into account to define the cross-reactivity, as DENV and WNV ratios were significantly lower than the JEV ratio. Plasma was also negative for IgM anti-CHIKV and anti-ZIKV (ELISA EUROIMMUN kits) (Table S1), as well as by PCR for CHIKV, DENV, ZIKV, and WNV (in-house assays on Panther Fusion, HOLOGIC) (see the supplemental material).

The patient was discharged from the hospital on 14 February (18 days after admission) with the persistence of partial aphasia and lack of coordination for fine gestures and reading difficulties. He presented with a partial seizure 10 days after discharge, and anticonvulsant treatment was introduced. A serum and a plasma sample were collected on the day of hospital discharge, showing a rise in anti-JEV IgG by the EUROIMMUN anti-JEV IgG ELISA kit, confirmed by seroneutralization.

Six months later the patient continues to demonstrate psychomotor retardation and word-finding difficulties. Blood tests showed the persistence of anti-JEV IgG and borderline IgM in serum. The urine samples tested negative by RT-qPCR. His wife’s serum sample, collected at the same time, was negative for both anti-JEV IgM and IgG.

## DISCUSSION

JEV RNA is rarely detected in blood or CSF samples, so the diagnosis of JEV infection is generally based on serological tests that can be challenging to interpret due to non-specific reactivity, cross-reactivity with other flaviviruses, and persistence following previous infection or immunization. Here, we identified two cases of JEV infection confirmed by RT-qPCR and seroneutralization, fitting the WHO definition of JE confirmed cases (WHO) (1).

The first patient traveled to Cambodia only, while the second one visited Vietnam first and then Cambodia. Although both countries have known JEV transmission, Case 2 likely acquired JEV infection in Cambodia (considering the delay between the stay in Vietnam and symptom onset). In Cambodia, JE is known as one of the leading causes of encephalitis, which affects mostly children (17, 18). The field studies on JEV infection and its circulation have concentrated on humans, pigs, and mosquitoes, revealing that JEV is widely prevalent across the country (19–21). Both patients visited very touristic areas in Cambodia, with documented JEV cases (Siem Reap and Phnom Penh) (18, 20); to our knowledge, there is no report of JEV circulation in Koh Rong Island so far.

JEV infection is associated with death and sequelae. A recent meta-analysis reported an 18% mortality rate and a 44% sequelae rate among symptomatic cases (22). In this report, neither patient reached full neurological recovery 6 months post-onset. Unlike Case 2 (71 years old), Case 1 had no high-risk conditions, such as immunocompromised status or extremes of age (23, 24), but experienced frequent mosquito bites, and neither patient was vaccinated against JEV.

For the majority of travelers to Southeast Asia, the risk of JEV is generally low, but it fluctuates according to specific factors such as the destination, duration of stay, season, and type of activities engaged in. The JE vaccine is recommended for travelers who intend to spend 1 month or more in areas where JEV is endemic during the transmission season (25, 26). Additionally, vaccination should also be considered for short-term travelers at increased exposure risk or those visiting areas with ongoing outbreaks. However, only an estimated 11% of eligible travelers receive the JE vaccine (27). Many travelers do not consult a travel clinic before departure, and among those who do, information about JE risk and severity is often insufficient (28). Travel health providers should be aware of potential life-changing consequences of JEV infection and provide travelers with clear information allowing them to make fully informed decisions on JE vaccination.

Detection of JEV RNA in clinical samples is rare and requires a highly sensitive molecular assay. We managed to confirm both cases by RT-qPCR, likely due to the patients’ early hospital admission shortly after symptom onset (days 4 and 1, respectively). Often JEV-infected patients consult a physician at the appearance of neurological symptoms when the acute phase is passed and the viral clearance has already occurred. Interestingly, we detected viral RNA in a urine sample from Case 1. To our knowledge, this is the third identification of JEV in urine and the second using RT-qPCR assay (the first detection occurred via deep sequencing) (16). In the second case report (14), the authors observed prolonged shedding of JEV in urine, up to day 26 after symptom onset; however, that patient also had viral RNA detected in whole blood. In our Case 1, urine was the only biological sample positive by PCR and allowed us to identify the etiological agent with certainty. Our findings are in contrast to a report from China, where JEV RNA was not detected by RT-qPCR from the urine of 52 children with serological evidence of JEV infection, collected from 3 to 9 days after disease onset (29). Another report from Laos (15) highlighted rapid viral RNA degradation in urine and proved that the immediate addition of lysis buffer to fresh urine improves the detection of JEV RNA at the limit of detection. The urine from Case 1 had not been diluted in lysis buffer and had not been tested immediately after collection since it was transferred to the French National Reference Laboratory; these aspects can also explain the high Ct values detected.

At the time of admission, both patients tested positive for anti-JEV IgM in blood and CSF samples, and interestingly, a long IgM persistence in serum has been observed for both cases (more than 6 months). There is very limited data on the detection of JEV IgM in serum following either natural infection or JE vaccination: one study reported that only 7% (3/41) of patients presented JEV-reactive IgM in serum >10 months after disease onset (30); additionally, after vaccination, the detection of IgM is extremely rare by 6 months (25).

Unfortunately, for both our cases, the attempt of viral isolation and genome sequencing was unsuccessful due to the very low viral loads; thus, we were not able to determine the JEV genotype responsible for these infections.

In this study, both patients experienced severe disease and required admission to the ICU, which highlights the importance of protective measures when traveling to a JE-endemic area, including JE vaccination and the use of personal measures to prevent mosquito bites (insect repellent, bed nets, long-sleeved shirts, and pants). Both patients had brief exposure to a JEV-endemic area and subsequently contracted the infection, underscoring the importance of thorough risk assessment. Patients can experience life-threatening illness with very serious consequences, even those considered not at risk for severe disease; when seeking travel health advice before the trip, travelers should be given information about the severity of JE to better inform their decision as to whether or not to be vaccinated.
